# Exploring Health Literacy in Individuals with Alcohol Addiction: A Mixed Methods Clinical Study

**DOI:** 10.3390/ijerph17186728

**Published:** 2020-09-15

**Authors:** Gabriela Rolova, Beata Gavurova, Benjamin Petruzelka

**Affiliations:** 1 Department of Addictology, First Faculty of Medicine, Charles University and General University Hospital in Prague, Apolinarska 4, 128 00 Prague 2, Czech Republic; gabriela.rolova@lf1.cuni.cz (G.R.); benjamin.petruzelka@lf1.cuni.cz (B.P.); 2 Faculty of Mining, Ecology, Process Control and Geotechnologies, Technical University of Košice, Letna 9, 042 00 Košice, Slovakia

**Keywords:** health literacy, alcohol addiction, HLS-EU-Q47, mixed methods, inpatient addiction treatment

## Abstract

This mixed methods research paper explores health literacy (HL) in individuals with alcohol addiction by using the 47-item version of the European Health Literacy Survey Questionnaire (HLS-EU-Q47) and semi-structured interviews concerning health-related competencies (access, understand, appraise, and apply health information), and determines the limitations of the HLS-EU-Q47 when used under specific conditions of clinical practice. The questionnaire survey and the interviews were conducted with individuals of different health literacy levels who were undergoing inpatient alcohol addiction treatment. The findings indicate that individuals with alcohol addiction might require different types of health information according to their health literacy level in terms of quantity and quality of information to recover from alcohol addiction and improve their overall health. The implications for the clinical practice of addiction treatment as well as recommendations for national and regional policy are also discussed.

## 1. Introduction

Health literacy (HL) has been identified as an important health construct with an impact on individual and population health [1,2,3]. According to the definition proposed by the European Health Literacy Consortium “Health literacy is linked to literacy and entails people’s knowledge, motivation and competences to access, understand, appraise, and apply health information in order to make judgments and take decisions in everyday life concerning healthcare, disease prevention and health promotion to maintain or improve quality of life during the life course” [4]. The relevance of this concept to public health has been further emphasized by national and international decision-makers in healthcare increasingly implementing health literacy into their health policies to promote health and quality of life [1]. 

In the past two decades, scientific interest in determining health literacy in people from diverse populations has been growing worldwide. The European Health Literacy Survey (HLS-EU), one of the first multinational surveys using a comprehensive approach to health literacy, revealed that nearly half of Europeans have limited health literacy and that there are substantial differences in health literacy between countries. Overall, the Czech Republic and Bulgaria were found to be the countries with the highest prevalence of limited health literacy [5]. In the Czech Republic, the national survey showed that nearly 60% of Czechs have limited health literacy [6]. Moreover, a following survey conducted in 2018 revealed that health literacy in Czechs was still decreasing [7].

Currently, research in the field of health literacy is becoming more and more concentrated on measuring health literacy in members of various specific population groups including people who use drugs. Overall, the findings indicate that health literacy in people with alcohol or substance use disorder is rather low [8,9]. Rolová et al. surveyed individuals undergoing treatment for alcohol dependence and found that up to half of the sample had limited health literacy [8]. The most recent study of Degan et al. examined the health literacy profiles of individuals who are undergoing addiction treatment and their relation to quality of life and mental and physical health. They identified 24.2% of individuals with low health literacy and 62.8% with moderate health literacy. The individuals with lower health literacy had worse social support, quality of life and mental health, and greater psychological distress [9]. Lincoln et al. examined the association between health literacy and severity of addiction in individuals undergoing medical detoxification. They identified 46% of individuals with low health literacy in inpatient detoxification units. However, an association between health literacy and severity of addiction has not been proven [10].

Health literacy was found to significantly affect health outcomes. Individuals with lower health literacy have worse physical and mental health status and a higher mortality rate [11,12]. Lower health literacy is further associated with worse access to healthcare, increased number of hospitalizations, lower use of preventive care, greater use of emergency care, poorer ability to understand medical materials, poorer medical adherence, and poorer knowledge of chronic diseases [11,13]. 

Moreover, the evidence suggests that health literacy might be associated with risky health behavior including smoking, alcohol consumption, and substance use [14,15,16,17], indicating that individuals with lower health literacy might be more susceptible to substance use and/or dependence [1,14,15,16,17]. Furthermore, there are possible additional health consequences for those with alcohol and substance use dependence, such as a greater risk of developing substance use-related diseases and complications (e.g. hepatic cirrhosis, skin and soft-tissue infections, intravascular complications, etc.), a risk of overdose, and further risky behavior such as sharing of injection materials, and hence a higher risk of infectious diseases [18,19]. Health literacy levels might also influence the protective effect of preventive programs and addiction treatment outcomes. Therefore, the consequences might include lower benefit from, or failure of, substance use prevention programs, worse access to addiction treatment programs, non-compliance with therapy, greater risk of drop-out, increased number of treatment repetitions, and greater susceptibility to relapse. However, evidence regarding health literacy in people who use drugs is still sparse. 

In this study, we aim to explore health literacy in individuals with alcohol dependence undergoing inpatient addiction treatment, by using a mixed methods research design. Our approach to health literacy consists of quantitative analysis in the form of the European Health Literacy Survey Questionnaire (HLS-EU-Q47) and qualitative analysis in the form of semi-structured interviews concerning health-related competencies elaborated by Sørensen et al. [4]. Furthermore, since this is one of the first studies using the HLS-EU-Q47 in a population of people who use drugs, we also focus on the understanding of the questionnaire from the perspective of individuals with different health literacy levels, different severity of alcohol addiction, and in different phases of addiction treatment. The question is whether the quantitative approach alone is a sufficient method to determine health literacy in individuals with alcohol addiction, or whether more frequent use of a qualitative approach should be considered, containing specifics for better understanding of the variables examined in the questionnaire survey.

Finally, the aims of the present study are: (1) to test the application of an integrated mixed methods research to clinical practice of inpatient addiction treatment in the Czech Republic, (2) to examine health literacy in individuals with alcohol addiction by using the HLS-EU-Q47, (3) to discover the limitations of the questionnaire in the clinical practice of inpatient addiction treatment, and (4) to explore differences in health-related competencies of participants with adequate and limited health literacy by using the semi-structured interviews.

## 2. Methods

### 2.1. Study Design

In this mixed methods study, we combined and integrated both quantitative and qualitative approaches [20,21]. We describe the mixed methods research design using Bryman’s criteria of priority and sequence [20]. The priority and first place in the data collection sequence was given to a quantitative approach and statistical analysis. The following qualitative component included semi-structured interviews and theory-driven approach to thematic analysis [22].

Bryman classifies mixed methods research based on how the two research approaches are used [20]. In this study, the following methods were employed:

(1) Quantitative research was used for sampling.
(2) Qualitative research was used for instrument development.
(3) The use of both research approaches contributed to the mutual development of the results (enhancement, augmenting).
(4) Both research approaches were used for mutual triangulation of the results.
(5) Both research approaches were used to explore unexpected results.

### 2.2. Participants and Data Collection

We used multistage sampling to select eligible participants for the study. 

Initially, the questionnaire survey was carried out to determine the health literacy level of the study sample and to identify the participants for the qualitative part of the study. The participants (*N* = 113) were men and women, 15 years and older, fluent in Czech, and undergoing long-term institutional treatment for alcohol dependence at the time of the data collection. 

Health literacy was measured using a Czech translation (official approval to use the Czech translation of the HLS-EU-Q47 was given by the National Institute of Public Health (Ref. PID UK1LF18G/03010 001)) of the HLS-EU-Q47. In a self-administered survey, the participants evaluated the perceived difficulty of health-related tasks on a 4-point Likert scale ranging from “very easy” to “very difficult” [4]. The reliability of the HLS-EU-Q47 was examined using Cronbach’s alpha. Internal consistency was high, with an alpha coefficient of 0.967 [8]. In addition, the validity and reliability of the HLS-EU-Q47 have been confirmed by several other studies in diverse populations [3,23,24,25]. A detailed methodology of the questionnaire survey, as well as complete results, are described elsewhere [8].

The semi-structured interviews were then carried out to explore the participants’ health-related competencies according to their health literacy levels determined in the questionnaire survey. Two-dimensional mixed methods sampling design, based on the sequential method of nested sampling defined by Onwuegbuzie and Collins, was used to select the participants [26]. Participants (*N* = 4) were selected from the baseline sample based on their score achieved in the HLS-EU-Q47. Two participants (a man and a woman) who reached the highest scores (43 and 42 out of 50, respectively) were selected as typical representatives of individuals with adequate health literacy and two participants (a man and a woman) who had the lowest scores (23 and 20 out of 50, respectively) were selected as typical representatives of individuals with limited health literacy. This strategy aimed to select these information-rich cases to contrast both ends of the health literacy continuum, to provide us with information on the extreme cases and to capture potential gender-specific differences manifested in health literacy. The support for this sample size comes from Malterud et al., who suggested that, while researching different aspects of a continuum, a smaller sample size of participants with opposite examined characteristics might be beneficial, with some limitations [27]. 

Furthermore, the criteria of Malterud et al. were used to evaluate the information power of the sample [27]. Our focus and aims were narrow and specific, and the sample was dense and well defined. Considering theoretical criteria, the study was based on existing theories and on addressing specific aspects (see below). Therefore, the specificity and the setting of this study required smaller sample size. Furthermore, the dialogue power of the interviews was evaluated by the research team as strong, since communication between the interviewers, who had good knowledge of the topic, and participants was evaluated as sufficiently clear (not ambiguous) and not conflictual. Furthermore, dialogue focused on the primary aim of the study, while providing enough information about the topic.

A self-generated identification code (SGIC) was used to identify the participants from the baseline sample. This anonymous identifier is generated by the participants based on their personal data that are unknown to the researcher. The code is a combination of characters (letters and digits) derived from elements (sources of information) [28]. The identifier was found to be a suitable tool with good results in terms of preserving anonymity regarding links to subjects [29]. The nine-character SGIC based on seven elements including name, surname, birth date, mother’s name, father’s name, paternal grandmother’s name and eye color of the participant was used [28]. First, all participants involved in the questionnaire survey filled in the questionnaires and the sheets with the SGIC. Then, the participants kept the sheets with the SGIC. The other part of the sheet with the anonymous code was delivered to the data collectors together with the questionnaire. Therefore, after the evaluation of the questionnaires, it was possible to identify participants for the qualitative part of the study by linking the codes with the individuals.

None of the participants who were selected refused to participate in the qualitative part of the study. The woman and the man with adequate health literacy are referred to as Participant 1 and Participant 2, respectively. The woman and the man with limited health literacy are referred to as Participant 3 and Participant 4, respectively. 

The semi-structured guide for the interviews was based on the HLS-EU-Q47. The interviews covered the general health-related task and alcohol-related tasks. The face-to-face interviews took place on 4 May 2018, 24 May 2018, and 11 June 2018, in one of the healthcare facilities for addiction treatment in Prague. Each of the four interviews was led by a different interviewer to prevent the interviewers from slipping into the same questioning patterns when interviewing the participants. The purpose of this procedure was to capture the issue in its entirety and from different perspectives. Finally, the 60-min audio-recorded interviews were transcribed by the same researchers who conducted the interviews.

### 2.3. Data Analysis

Data analysis consisted of statistical analysis of the questionnaires and thematic analysis of the interviews. 

A general health literacy (GHL) index determining participants’ health literacy levels was calculated together with specific indices of health literacy in three subdomains of the questionnaire—healthcare, disease prevention, and health promotion. The following formula was used to calculate the indices of the GHL and individual domains: Index = (mean − 1) × (50/3), where Index is the specific index calculated, mean is the mean of all participating items for each individual, 1 is the minimal possible value of the mean, 3 is the range of the mean, and 50 is the chosen maximum value of the new metric. Index 0 represents the lowest possible health literacy and 50 the highest health literacy [30]. Health literacy levels were defined as “inadequate” (0–25), “problematic” (>25–33), “sufficient” (>33–42) and “excellent” (>42–50). Inadequate and problematic levels determine “limited health literacy” and sufficient and excellent levels determine “adequate health literacy” [4]. The quantitative data were analysed by using IBM SPSS Statistics 23 (IBM, Armonk, NY, USA). 

The interviews were analysed using a theory-driven approach to thematic analysis. This approach was used as a framework for coding and analytical process. A combination of open and structured coding was applied. 

First, four basic frame codes—access, understand, appraise, and apply health information—were created to explore the health-related competencies of the participants. The frame codes corresponded to health-related competencies defined in the conceptual model of health literacy. “Access refers to the ability to seek, find and obtain health information, understand refers to the ability to comprehend the health information that is accessed, appraise describes the ability to interpret, filter, judge and evaluate the health information that has been accessed, and apply refers to the ability to communicate and use the information to make a decision to maintain and improve health” [4]. Second, individual codes corresponding to the individual items in the HLS-EU-Q47 were created. However, not all health-related tasks were covered in the interviews as the primary focus was on problematic tasks identified in the questionnaire survey. An additional code concerning the understanding of the HLS-EU-Q47 was created to find the limitations of the questionnaire utilization when used under specific conditions. 

The data were manually coded by a single researcher and then revised by another researcher to enhance data validity. Based on the coding, the reports on the themes were produced.

### 2.4. Research Team

The research team consisted of several interviewers and researchers. There were four university-educated interviewers (three women and a man) in the research team, each conducting and transcribing one interview. The interviewers were experts in addiction sciences and had experience of qualitative research. Before data collection, the interviewers were educated in the concept of health literacy and trained in conducting and transcribing the interviews by the researchers. The field notes were taken during and after completion of each interview.

Two researchers in doctoral training of addiction sciences (a man and a woman) with education in qualitative research supervised the collection and transcription of the data and provided the coding, analysis procedure, and interpretation of the data (for detailed contribution of the authors, see below). 

There was no prior relationship nor close contact between the participants and interviewers/researchers until the data collection took place. The participants and interviewers/researchers did not exchange any personal data except for those stated in this article. 

### 2.5. Ethical Approval

The design and realization of the study were approved by the Review Board of the General University Hospital in Prague (Ref. 101/17 grant GAUK 1. LF UK). All participants were informed of the study purpose and signed an informed consent regarding participation in the study. This study was conducted with respect to the seventh revision of the World Medical Association Declaration of Helsinki [31] and the second revision of the Farmington Consensus [32].

## 3. Results

The participants’ sociodemographic characteristics are presented in Table 1. The participants with adequate health literacy were university educated, self-employed, and belonged to a higher economic and social strata. The woman with adequate health literacy graduated in Medicine and had worked as a private dentist. The participants with limited health literacy were secondary school graduates and belonged to the middle and lower social strata. Three participants (all except for Participant 4) declared having additional mental health issues on top of alcohol dependence.

### 3.1. Quantitative Analysis

The complete scores of the participants in GHL, healthcare, disease prevention, and health promotion domains, as well as the individual scores in the four competencies of health literacy, are shown in Table 2.

Overall, the woman and man with adequate health literacy achieved 42 and 43 points (out of 50) indicating sufficient and excellent general health literacy, respectively. The woman with adequate health literacy reached an excellent level in the healthcare domain but she scored one level lower (sufficient) in the other two domains. The man with adequate health literacy achieved more stable results with the lowest score in the healthcare domain. The woman and man with limited health literacy achieved 20 and 23 points indicating inadequate general health literacy. The woman with limited health literacy scored low in all three domains. The man with limited health literacy achieved borderline scores in healthcare and health promotion domains but his score was much lower in the disease prevention domain. 

With regards to health-related competencies, the participants with adequate health literacy reached at least a sufficient level in all health-related competencies and achieved the highest scores in understanding health information. The woman with adequate health literacy achieved the lowest score in accessing health information while the man with adequate health literacy achieved the lowest score in appraising health information. The participants with limited health literacy mostly reached inadequate levels in health-related competencies. Both achieved the highest scores in understanding health information and the lowest scores in appraising health information. 

From the participants’ point of view, the most difficult health-related tasks were to “find information on how to manage mental health problems like stress or depression” (no. 18) in accessing health information, to “understand information on how to keep their mind healthy” (no. 40) in understanding health information, to “judge when they may need to get a second opinion from another doctor” (no. 11) in appraising health information, and to “decide how they can protect themselves from illness based on information in the media” (no. 31) in applying health information.

The overall results of the questionnaire survey of health literacy in people who are undergoing treatment of alcohol disorder are presented in the original article of Rolová et al. [8].

### 3.2. Qualitative Analysis

#### 3.2.1. Circumstances of Alcohol Abuse 

To provide the context for further results, the participants’ description of their alcohol problems is introduced. 

The participants with adequate health literacy had started to use alcohol during their secondary education. What was initially social drinking turned into alcohol abuse during their adulthood due to long-term stress exposure and occurrence of anxiety spectrum disorders. The woman with adequate health literacy (Participant 1) reported that her alcohol drinking peaked in her forties due to mental issues arising from partnership issues which she tried to alleviate with the combination of alcohol and sedatives. She was undergoing her second long-term institutional treatment after almost 20 years of remission at the time of the study. The man with adequate health literacy (Participant 2) stated that his alcohol drinking turned into abuse due to anxiety associated with his work responsibilities. He was undergoing his first long-term institutional treatment at the time of the study. Both participants agreed that alcohol drinking provided them with relief and helped them to alleviate anxiety, which indicates that they were unable to cope with stress exposure effectively. They both stated that they prefer low-alcohol beverages. 

The participants with limited health literacy started to abuse alcohol soon after their first experiences. The woman with limited health literacy (Participant 3) stated that she started drinking larger doses of alcohol in response to a lack of energy caused by anorexia and bulimia from the age of 17. Later, she developed alcohol dependence due to stress exposure associated with childcare responsibilities. At the time of the study, she was undergoing her second long-term institutional treatment. The man with limited health literacy (Participant 4) reported frequent drinking because he was enjoying the state of altered consciousness associated with alcohol intoxication. He was undergoing his fourth long-term institutional treatment at the time of the study due to repeated relapses. Both participants stated that they prefer liquors and spirits and felt more relaxed after alcohol drinking.

#### 3.2.2. Understanding of the HLS-EU-Q47 

In general, all participants reported a good understanding of the individual items in the HLS-EU-Q47. However, limitations related to the utilization of the questionnaire in clinical practice were found. In particular, the participants discussed the length of the questionnaire, the context of the questions and the general focus.

The woman with adequate health literacy expressed her concerns about the length of the questionnaire in connection with her feeling of fatigue and exhaustion stemming from a demanding therapeutic program, as she was at the very beginning of the addiction treatment:

*“...as I did not get familiarized with the atmosphere yet. It seemed long to me (the questionnaire) because I was scared of adhering to the demands of the program.” [Participant 1]*

The woman with limited health literacy pointed out that it was difficult for her to evaluate the perceived difficulty of many of the health-related tasks in the questionnaire as she did not encounter those situations in practice:

*“Well, I did not encounter some questions in practice. I could have rated them, but they were not from practice. It was more theorizing... They were very hypothetical (the health-related tasks), and I had a lot to guess what my reaction might be” [Participant 3]*

The man with limited health literacy also reported a good understanding of the questionnaire but was puzzled by its general focus, which made it difficult for him to answer the individual questions:

*“There were no problems at all. What seems strange to me was that the questionnaire had nothing to do with my illness (alcohol dependence). It (the questionnaire) was general. So, it seems strange to me. I asked if I should answer the questions in connection with my disease or in general. Because obviously there is a difference whether one should seek medical help due to flu or because he is an alcoholic. So, this was not clear to me.” [Participant 4]*

#### 3.2.3. Accessing Health Information 

All participants answered positively to the question asking if they knew how to access health information and where to find medical help. However, important differences between both groups were found in terms of preferred sources of information and efforts to access information.

The participants with adequate health literacy reported actively seeking information about their health problems in various sources including the medical literature and via web search. However, despite their active approach to accessing health information, they still rely on healthcare providers as the main sources of health information. Furthermore, they were confident in discussing health-related questions with healthcare providers and participating in their treatment:

*“I trust doctors because they are experts. I am still just a layman, even if I can get some information by myself. In case, it is something extra serious, one is eagerly trying to get some information about it. For example, in the case of life-threatening surgery, if I presented it as a model situation, I would be probably consulting it with more than one doctor.” [Participant 2]*

The participants with limited health literacy, on the other hand, reported usually relying on healthcare providers as sole source of information. They were rather passive recipients of health information as they did not actively seek it in other relevant sources. However, the statement given by the man with limited health literacy about his interest in acquiring health information suggests that they might not know where to look for such information:

*“Did you yourself were looking for information about your illness (alcohol dependence) in other sources? Somewhere outside of healthcare providers, medical professionals, nurses?” [Interviewer] “Not exactly, but I would definitely want to, and if there are any publications, I would like to learn something.” [Participant 4]*

Interestingly, except for the woman with medical education (Participant 1), the participants had a critical attitude towards seeking health information on the web:

*Well, I am definitely not one of those who determine the diagnosis themselves because of the Internet... Well, either there might be nonsense, but there might be also the things that might be true. But if it was that simple, the medical professionals would not have to study for six years and then make the specialization.” [Participant 2]*

The differences observed between both groups of participants also applied in the case of accessing information about alcohol and dependence. Whereas the participants with adequate health literacy actively sought alcohol-related information from various sources during the treatment process, the participants with limited health literacy relied on information given by healthcare providers only:

*“When I am sick, I go to see an expert and wait for what they tell me. I do not understand it at all, I will not make up things that are not what they say. When they say it is like that, it is just like that.” [Participant 4]*

#### 3.2.4. Understanding Health Information 

Overall, all participants claimed to understand health information at least well enough to manage their health problems. However, when they were further questioned, we found that the level of understanding varied between the groups. 

The participants with adequate health literacy reported a good understanding of health information including the information provided by healthcare providers, written materials for the general public, and books popularizing medicine. Moreover, the participants with adequate health literacy frequently used various medical terms (e.g. dehydration, serotonin, lymphatic) during the interviewing, whose meaning they seemed to understand.

*“Do you understand the provided information (by the healthcare providers)?” [Interviewer] “Yes I most certainly do, and in case I had not understood something, I have asked, and it was explained to me.” [Participant 2]*

The participants with limited health literacy also stated that they understood various common forms of health information such as that provided by their healthcare providers, patient information leaflets, and prevention materials. However, as the man with limited health literacy stated, he often does not understand the context as he has no medical background, which does not allow him to fully cooperate with healthcare providers:

*“I take it like it is their job and these people are experts who know what they are doing. I do not try to say to them that they do not know something or that they do it wrong, because nobody will tell me such a thing in my work. I am not an expert and I do not understand it, so I take it as it is, and it should be always like that. I will trust them (the healthcare providers). What should I say to them when I do not know anything about it?” [Participant 4]*

Interestingly though, all participants reported that they usually ask healthcare providers for further explanation if they have difficulty understanding the provided health information, suggesting that they have strategies for how to cope with gaps in their competencies:

*“So, you usually consult it (use of medication) with the healthcare provider.” [Interviewer] “Yes, I do. I always ask if there is any chance of intolerance, if I can take it with my other medication, before or after a meal and so on.” [Participant 3]*

When questioned about their understanding of alcohol-related information, all participants reported a good understanding of information concerning the health consequences of excessive alcohol drinking, the nature of alcohol dependence, and the principles of the addiction treatment process. However, as the man with limited health literacy described, his knowledge and understanding of alcohol-related information evolved over time with a significant contribution from addiction treatment programs, suggesting their important educational component.

*“I know a lot about it (alcohol addiction) because I have been an alcoholic for a long time. I did not even know what being an alcoholic means... in my youth.” [Participant 4]*

#### 3.2.5. Appraising Health Information

In general, all participants reported being able to assess their health condition and the severity of disease symptoms. Moreover, the participants were aware that their lifestyle affected their health outcomes. However, both groups differed significantly in the amount of confidence in their ability to appraise health information and the extent to which they were able to use their knowledge and experiences to appraise health information.

The participants with adequate health literacy showed higher confidence in their ability to appraise health information. They also reported being able to use their knowledge to appraise health information such as the severity of acute health problems:

*“One has some general knowledge about common illnesses like flu, virus-like diseases or rhinitis, headache... So, in my years, when I am already 35 years old, one has some awareness of what he can do alone and what he cannot solve. Of course, if I had some abdominal pain, I would seek medical attention. Nevertheless, at that moment when I have a temperature of 38.5 degrees Celsius, at this moment, I do not seek information, I will attribute it to the flu, go to the pharmacy and buy myself the Paralen or Brufen. Of course, at a time when it would not improve for a long time, I would probably find out that it is not the flu, and then I would deal with it.” [Participant 2]*

Furthermore, the participants with adequate health literacy stated that they are usually cautious concerning any health information from the media as they are aware that the information might not be credible. They were also able to evaluate living and environmental conditions affecting their health outcomes:

*“We live in a relatively small village... Those conditions are probably not ideal, because it is still relatively close to Prague. I must admit that I do not monitor any concentrations that occur there, whether allergens or toxic compounds, but I think that as for the Czech Republic, it is above standard...” [Participant 2]*

In addition, the participants with adequate health literacy were also able to evaluate the benefits and negatives of different types of treatment facility:

*“I have a comparison with another treatment facility. I have to say that there is the more individual approach here and what is very different is that here you have a responsibility both for yourself and for the team, which is not the case in the other treatment facility.” [Participant 1]*

In contrast, the participants with limited health literacy showed low confidence in their ability to appraise health information as they were aware of their inadequate health-related knowledge and experiences. Therefore, as the participants reported, they usually rely on the judgement of others such as healthcare providers or more knowledgeable acquaintances, as concerns the appraisal of health information:

*“When the children were small and when I did not want to annoy my pediatrician anymore, I used to call one of our family acquaintances several times to make sure that something was good or bad.” [Participant 3]*

Furthermore, participants with limited health literacy seemed to have trouble evaluating health information in a broader context. For example, the man with limited health literacy was aware that his lifestyle and living conditions might affect his health outcomes, but he evaluated their effect at a very basic level, not considering the complexity of this issue.

*“I lived in the fodder rack in the woods for one time and it was cold, and these things are not good. But because I was that old as I was then, I survived. Anyway, a one needs to have warmth, dryness and a place to wash. This is important to me in terms of health.” [Participant 4]*

Regarding the appraisal of alcohol-related information, all participants were able to assess the health risks of alcohol drinking in general and in connection with their health condition:

*“What I am saying, and what really bothers me is that it is so terribly benevolent here. It is dangerous when alcohol can be bought 24/7. It is dangerous and for me for sure.” [Participant 4]*

However, the participants with adequate health literacy seemed to have more comprehensive knowledge about this issue as they specified concrete health problems and used more expert terms:

*“What about that brain... I definitely have worse memory, but it should be reversible. Well, we will see, it is still a short time. Certainly, it (alcohol drinking) can negatively affect the pancreas, digestive tract... And the body, of course: I was dehydrated, demineralized, so it definitely has an impact on joints and teeth... It affects the whole organism.” [Participant 2]*

Interestingly, both groups expressed their concerns about the benevolent alcohol policy of the Czech Republic:

*“I just say that on every single cigarette pack is written that smoking kills, but they will not write on alcohol bottle what are the consequences of booze. It is because we are a pro-alcoholic government.” [Participant 3]*

#### 3.2.6. Applying Health Information 

Overall, all participants reported being able to apply the health information given by their healthcare provider in order to improve their health when they have any problems. However, a difference concerning compliance and adherence with treatment recommendations was found between the groups. Moreover, gender-specific differences were found in terms of applying health information in order to improve lifestyle. 

The participants with adequate health literacy seemed to be able to use health information to make decisions, based on their ability to appraise health information in order to improve their health condition. They claimed they usually have no trouble in following health recommendation to reduce the risk of developing health problems:

*“...I have always read the patient information leaflets and tried to follow them. In particular, I pay attention to how much it affects concentration while driving, to be sure not to produce an accident. And I look at the counter-indications, of course, especially so that the drugs do not cross each other, so I pay a lot of attention to that.” [Participant 2]*

The participants with limited health literacy described some experience of non-compliance with the treatment process, as they sometimes purposely did not follow the instructions of the healthcare providers:

*“You suffer from neuropathy, for example. Is he (medical professional) able to explain to you in a way you understand what you are suffering from and what is the cause?” [Interviewer] “I understood this very well. Another question is if I follow it then.” [Participant 4]*

The woman with limited health literacy admitted that she does not read patient information leaflets, suggesting the possible risk of medication misapplication. However, she also described an alternative strategy:

*“I do not read them (patient information leaflets), but if I must take something, I ask him (healthcare provider) directly about what I am interested in.” [Participant 3]*

Furthermore, as the participants described, the men were not interested in healthy eating and smoked cigarettes daily even though they were aware of the impact of lifestyle on health. The women, on the other hand, were aware of the principles of a healthy lifestyle especially regarding a healthy diet and its impact on health and avoidance of cigarette smoking, and they tried to adhere to those principles daily:

*“...I try to eat adequately with my age because you do not eat as 20-year-old when you are older, and I think I have the ideal weight.” [Participant 2]*

However, both men and women agreed on the important role of sports and exercises for health and claimed to participate in sport regularly:

*“I currently smoke and undergo addiction treatment for alcohol addiction. This means that I am the very last person that would say that he is striving for a healthy lifestyle... So, I smoke, that is true, but I do a lot of sports and this extremely helps the body.” [Participant 2]*

Concerning the application of alcohol-related information, all participants claimed that they were determined to abstain from alcohol, based on their knowledge and experience acquired during the addiction treatment. The participants also reported being able to adhere to the addiction treatment program and to apply the acquired experience in order to prevent relapse. However, as the addiction treatment history of the participants with limited health literacy showed, even though they were equipped with knowledge and strategies on how to prevent relapse, they failed several times to do so.

*“I entered (the addiction treatment) with some expectations. Firstly, I would fix the mess I did, secondly, find the reason why the relapse actually happened to me and what I actually had in my head, and thirdly, why the crisis plan did not work. The one when he is out (of treatment) and drinking alcohol, should be back in here very fast. But I, for my sake of stubbornness, made it all more complicated for me...” [Participant 3]*

## 4. Discussion

In this study, health literacy of individuals with alcohol addiction was examined using the mixed methods approach. The aim was to explore the differences in health-related competencies in men and women with adequate and limited health literacy. Moreover, the understanding of the Czech version of the HLS-EU-Q47 under specific conditions of clinical practice was explored. 

The findings suggest that the mixed methods approach is a convenient method to explore this phenomenon from multiple perspectives. Moreover, the combination and integration of both quantitative and qualitative approaches allowed the determining of participants’ health literacy and find out about distinctions in health-related competencies that exist between individuals with adequate health literacy and limited health literacy. 

Using the qualitative approach, very specific knowledge of the health-related competencies of the participants was obtained. However, the findings from qualitative analysis were not conclusive in themselves as regards to the participants’ level of health literacy. The quantitative analysis, on the other hand, provided more unequivocal information about the participants’ level of health literacy. Therefore, the advantage of the mixed methods approach lies in its complementarity and specificity. The combination of quantitative and qualitative approaches increases the understanding of this issue, still very little examined contemporarily.

### 4.1. Understanding of the HLS-EU-Q47 

In this study, the participants reported general satisfaction with the understanding of the questionnaire. However, we found minor limitations which might affect the outcomes of the questionnaire survey related to the clinical setting of the questionnaire administration and semantic effects. Therefore, these limitations should be taken into account when designing a study [34]. 

First, the specifics associated with the rules of the treatment program were identified. In long-term addiction treatment (e.g., the Apolinar or Skála addiction treatment models combine the principles of a therapeutic community with behavioral approaches [35]), patients must adhere to a very tight schedule and scoring system. This is more problematic for individuals at the beginning of treatment as was reported by one of the participants. It can be assumed that stress from a new environment might result in reduced attention when completing the questionnaire. It may be suggested that better timing and submission of the questionnaire in later phases of the treatment might produce more accurate results. In addition, there are other relevant specifics of the population under study, such as the use of certain medication or the factors related to current health condition including mental health issues. Thus, attention should be paid to the selection criteria for the individuals participating in the study. The examination of factors such as the degree of health impairment or the length and severity of disability might significantly improve the quality of data which would produce more accurate results in future research.

Second, limitations based on semantic effects were found. According to the Tourangeau et al., there is a necessity to convey an intended semantic space which the respondent reconstructs [36]. In this case, it was difficult for the participants to reconstruct this if the intended space is oriented towards their disease (alcohol addiction) or to other general health problems. Thus, for these specific populations, it is important to address these uncertainties at the beginning of the interview. Furthermore, one of the participants pointed out that the specific questions were “theoretical” and required hypothetical considerations, meaning that they were not well defined. This indicates that the answers used in the questionnaire might be too general and thus provide very different stimuli for different participants.

In conclusion, minor adjustments of the HLS-EU-Q47 based on the specifics of the study population and minimalization of the effects stemming from the clinical setting of the survey might be necessary to increase the validity of the data. It may be assumed that the differences in health literacy in participants could have influenced their perception of the questionnaire and the relevance of their responses as well as their understanding of the questionnaire. We believe especially that individuals with lower health literacy might benefit from the questions being customized to the situations they encounter in everyday life. However, it should be noted that the readability of the Czech version of the HLS-EU-Q47 was not tested, nor was the reading level of the participants. Therefore, it is not possible to conclude whether the Czech translation of the HLS-EU-Q47 is suitable for those with lower health literacy. As Degan et al. found out, individuals with low health literacy who are undergoing addiction treatment reported significantly lower reading ability than individuals with higher health literacy [9]. The suitability of the short version of the questionnaire (HLS-EU-Q16) for individuals with limited literacy was investigated by Storms et al. They found the HLS-EU-Q16 to be suitable for individuals with limited health literacy but concluded that minor improvements would be beneficial [37]. However, it may be assumed that in the case of the HLS-EU-Q47 version, the length of the questionnaire may also play an important role in its understanding. Finally, these findings must be interpreted with regard to the findings of Trujols et al., who found that patients claiming to be highly satisfied with treatment generally provide different perspectives when asked more specifically [38].

### 4.2. Health Literacy Competencies

Overall, it may be confirmed that the participants’ health-related competencies explored by the qualitative approach correspond to their level of health literacy determined by the HLS-EU-Q47. 

Interviews showed that participants with adequate health literacy had a better ability to evaluate health information critically, were more interested in their health condition and health-promoting activities, seek health information, and had better communication skills. Furthermore, they were able to use and understand medical terminology and utilize their health-related knowledge in practice. 

Interestingly, it was found participants had various strategies to deal with insufficient health literacy competencies. ‘I will ask the doctor’ was the most universal strategy the participants used when they had difficulties in accessing or understanding health information. This indicates that the differences between those with low literacy and high literacy may not be so abysmal, but only that those with limited health literacy use different strategies in terms of health-related competencies that may be more or less effective.

### 4.3. Accessing Health Information 

As for accessing health information, participants with adequate health literacy reported using various sources of health information and an active approach to information finding including searching the Internet. On the contrary, participants with limited health literacy were not interested in these health-related tasks. This finding is in line with that of Von Wagner et al., who found that lower health literacy is associated with finding less information, which can be partially explained by the fact that individuals with lower health literacy must put a greater effort into processing written information, as the authors further explain [39]. 

In participants with adequate health literacy, information finding was closely associated in our study with critical thinking and trust in medical professionals. Tsai et al. found health literacy to be significantly associated with trust in medical professionals and in the healthcare system [40]. However, Aboumatar et al. argue that individuals with lower health literacy report a similar level of trust in medical professionals as participants with higher health literacy [41]. Interestingly, participants with limited health literacy more often perceived the medical professional as the sole source of information. This finding is supported by Wolf et al. who reported that HIV patients with limited health literacy were more likely to use their medical professional as a primary source of information [42].

Searching for health-related information might expand knowledge about the issue of interest and support the self-management of health problems, which is particularly important as the time availability of healthcare providers is limited.

### 4.4. Understanding Health Information 

As for understanding health information, the participants claimed to understand the information and instructions provided by their healthcare providers. This is supported by the analysis of the HLS-EU-Q47 as all participant achieved the highest scores within this competency. However, it was found that the degree of understanding and complexity of information they can understand is the point at which participants with adequate health literacy differ from those with limited health literacy. Participants with adequate health literacy reported a better understanding of complex health and medical information and were able to understand the impact of risky health behavior on their health. On the other hand, participants with limited health literacy reported understanding of basic health information in terms of how to manage their health problems. This is in line with the findings of Degan et al., who found out that ‘understanding health information well enough to know what to do’ was the item (in the Health Literacy Questionnaire, HLQ) where individuals with low health literacy scored the highest [9].

In addition, participants with adequate health literacy were able to use and understand medical terminology whereas participants with limited health literacy mostly used plain language when talking about health matters. It is assumed that the use of medical terminology might be an important indicator of health literacy level. Support for this assumption comes from the fact that one of the most widely used instruments measuring functional health literacy—the Rapid Estimate of Adult Literacy in Medicine, REALM—is based on the pronunciation of various medical terms [43]. The use of medical terminology might promote communication with the medical professional at a higher level and be beneficial for both sides. However, healthcare providers should be aware of the patient’s ability to understand medical terminology, otherwise, this might lead to misunderstanding with possible health implications.

Sørensen et al. [4] stated that understanding health information is an important premise not only for comprehension of instructions provided by the healthcare provider but also for comprehension of the implications of daily behaviour in relation to health. The findings from this study indicate that understanding health information is also an important premise for appraising health information and for decision making. However, participants with limited health literacy achieved the lowest scores in appraising health information despite their highest scores in understanding health information in the HLS-EU-Q47. This discrepancy might indicate that their limitations in understanding health information are more serious than they are able to evaluate.

### 4.5. Appraising Health Information 

As for appraising health information, participants with adequate health literacy were able to appraise health information on how to maintain and improve health comprehensively, and in the broader context of other relevant information. On the contrary, participants with limited health literacy were very uncertain about their ability to appraise health information and its credibility and, rather, relied on someone else’s opinion. Moreover, they seemed to appraise health information in a narrower context without considering other related information. The difficulties with appraising health information reported by the participants with limited health literacy are in line with the analysis of the HLS-EU-Q47 as they both achieved the lowest scores (14 and 11 out of 50) within this competency.

In conclusion, the ability to appraise health information turned out to be one of the most important competencies for maintaining and improving health. However, it is also the most difficult task for the participants, as three of them achieved the lowest scores within this competency.

One of the important aspects within this competency is the ability to evaluate the credibility of health information. The number of people seeking health-related information online via the Internet is increasing [44]. However, the quality of the information varies and a considerable proportion may be considered as misleading or false [45]. Thus, the ability to distinguish between good and bad information might be crucial for maintaining and improving health. As reported by Champlin et al., individuals of all levels of health literacy have difficulties in assessing which information is credible and which is not. This is particularly true for online information and for recognition of whether the information found is for marketing purposes only or if it has real value [46]. Diviani et al. argue that health literacy is positively associated with trust in online health information sources and the ability to evaluate online health information [47].

### 4.6. Applying Health Information 

As for applying health information, participants were able to follow instructions from healthcare providers concerning the maintenance of their health and treatment process. However, participants with limited health literacy were limited in applying health information by problems with the other two competencies—understanding and appraising health information. As one of the participants with limited health literacy mentioned, he sometimes does not follow the treatment instructions intentionally. Thus, the question is not whether the individuals can apply health-related information but whether they want to apply their knowledge. Miller reported that patients with a lower level of health literacy have lower rates of adherence than patients with a higher level of health literacy [48].

Non-compliance with treatment can be particularly problematic for managing illness that does not manifest externally, but where the risk is all the greater if the treatment is not properly followed (e.g. diabetes, high blood pressure). This kind of health issue often brings various barriers to everyday life, which a patient cannot always accept. Moreover, the mastering of this competency might be important, as shared decision-making has an important position in contemporary healthcare.

In addition, a difference was found between men and women in terms of applying information concerning healthy lifestyle and avoiding risky behavior (e.g. cigarette smoking) to improve health. This suggests that women are more interested than men in disease prevention influenced by lifestyle. The support for this hypothesis comes from Divine and Lepisto who found that women tend to be more interested in a healthy lifestyle than man [49].

In terms of alcohol addiction, the competencies of the participants were more comparable. The participants reported deep knowledge about alcohol addiction including the nature of the addiction itself and recovery and were even able to understand medical information related to addiction and to judge this. However, participants with adequate health literacy were more interested in accessing alcohol-related information, seemed to understand alcohol addiction in a broader context, and were relatively more successful in managing alcohol addiction and related problems.

Besides the differences in the four competencies of health literacy, there were also differences in health literacy and sociodemographic characteristics (educational attainment, household net income) and the severity of the mental health problems of the participants. Participants with limited health literacy had more severe alcohol problems in terms of number of relapses and treatments. Furthermore, the woman with limited health literacy suffered from long-term bulimia nervosa, a condition closely related to addictive behavior. The association between health literacy and mental health has been investigated in the literature. Wolf et al. found that older adults with limited health literacy had worse mental health than individuals with adequate health literacy [12]. Lincoln et al. reported that adults with low literacy and addiction suffer from more depressive symptoms than highly literate individuals. However, they did not find any significant association between health literacy and severity of alcohol addiction [10].

### 4.7. Limitations

Limitations should be acknowledged in interpreting the findings of this study correctly. 

First, the design of this study is very specific in terms of population, clinical setting, and the utilization of the HLS-EU-Q47. This instrument was originally designated for measuring health literacy in the European general population [3]. However, the research team evaluated the use of this instrument as appropriate for its own purposes. Moreover, since the HLS-EU-Q47 is increasingly used in specific populations and settings [24,50,51], the findings from this study might be especially beneficial for those who are using the HLS-EU-Q47 for similar purposes.

Second, the qualitative findings are based on the statements of four participants. The small sample size limits the generalization of the results but does not reduce their informative value. The very narrow and specific aim of this study required a smaller but dense sample to avoid loss of an important part of the information in the data since the aim of this study was to introduce the study topic. The implications for the practice of inpatient addiction treatment are also discussed (see below). In addition, it is important to note that no control group was established in this study. 

Third, the quantitative findings are based on detailed analysis of HLS-EU-Q47 answers of four participants. Quantitative analysis was conducted in order to determine the participants’ level of health literacy, identify their health-related competencies in relation to their level of health literacy, and compare the findings of the questionnaire with findings of the interview. However, it is not possible to draw any general conclusions from the results based on the analysis of such a small sample.

Finally, the data were obtained from participants with different experience and severity of alcohol problems, and in different phases of addiction treatment. Hence, longitudinal aspects of this issue were not captured. Health literacy in participants is likely to change over time and experiences with the addiction treatment process play a role. Further research should focus on the comparison of the health literacy in individuals at the very beginning of the treatment, during the treatment, and at its end.

Moreover, the literal transcripts were not returned to the participants for their approval as the interviews were very well understood.

### 4.8. Implications for Practice

These findings are important for clinical practice of addiction treatment in terms of improving treatment outcomes of individuals as well as their overall health. The findings indicate that individuals who are undergoing treatment for alcohol addiction with different levels of health literacy might benefit from different approaches and different types of information in terms of quantity and quality. This brings us back to the need for individualized medicine, which is currently a major challenge for psychiatric care, including addiction treatment, due to different disease manifestations and treatment responses [52].

The findings further suggest that the ability to apply health information might play an important role in the recovery from alcohol addiction. Although the participants showed a great deal of knowledge about alcohol addiction and the ability to understand and appraise alcohol-related information, they had issues with applying this information when relapse occurred. In the case of abstinence violation, all learned strategies on how to prevent relapse began to fail. This indicates that patients might be able to gain a great deal of alcohol-related knowledge in the inpatient addiction treatment, but not necessarily gain competencies. Hence, there needs to be a focus on empowering patients in this particular competency.

In general, attention should be also paid to the role of primary prevention and healthcare in health literacy. The primary healthcare professionals are the first contact points of patients with the healthcare system. Thus, they play an important role in patient orientation. A satisfactory estimate of health literacy in patients in primary care and the provision of adequate information according to health literacy level may have a crucial role in the further healthcare process.

Moreover, for elderly patients and individuals living in less accessible areas, the primary healthcare professional is their only health contact. Therefore, the primary healthcare professional should not only show a quantitative outcome in the form of a cured patient but also a qualitative outcome in an increase in health literacy of those who need it. Further research should explore the geographical disparities in health literacy in relation to quality and availability of healthcare. Knowledge of this context will greatly contribute to improving health indicators in the region.

Furthermore, the findings from similar studies are also important for health and social policymakers across multiple fields. The current lack of interest of national and regional policymakers in health literacy, whose exploration is at the forefront of current health policy, is reflected in the absence of quality prevention programs and their transformation into national and regional strategic plans.

This study declares that its outputs are relevant both for health and social policy and also for the methodological and conceptual field of study. This is related to the improvement of research interviewing techniques and support tools, and the detection of limitations in the collection of sensitive data for individuals with specific health problems that are highly individualized due to the impact of health and socioeconomic aspects. This creates a platform for national as well as international benchmarking and the active sharing of research knowledge by international multidisciplinary teams. The effect of these processes will be evident along a number of lines, including a wider diagnostic research focus, and support for follow-up research towards one goal—to permanently support public health and to improve the quality of life of the disabled population in order to eliminate health disparities that are the concern not only to the EC but also other global international institutions.

## 5. Conclusions

The findings from this study provided insight into alcohol addiction from the health literacy perspective. While the quantitative results described the overall level of health literacy and highlighted differences in competencies related to health literacy, the qualitative analysis captured other aspects that were not possible to derive from statistical analysis of the questionnaire. Similar studies measuring health literacy under specific conditions might benefit from the presented limitations of the HLS-EU-Q47.

A platform for further research has been created, in order to answer the question of whether the HLS-EU-Q47 is appropriate to determine health literacy in specific populations, or whether its utility may vary according to the type and degree of morbidity (alcohol addiction, mental illnesses, non-communicable diseases, etc.). Hence, a subsequent question is whether the degree of mental health or disability of the respondents might affect the choice and combination of research methods.

Future research should focus on further improvement of the instruments measuring health literacy in specific population groups, by both the quantitative and qualitative approach, and suggesting interventions for improving health literacy in the general population, as well as in specific population groups. Moreover, it is believed that the implementation of the health literacy concept in the national and regional politics of the European countries is a necessary step to improve population health.

## Figures and Tables

**Table 1:** Sociodemographic characteristics of participants.

HL	Participant 1	Participant 2	Participant 3	Participant 4
	(Adequate)	(Adequate)	(Limited)	(Limited)
Gender	female	male	female	male
Age	61	35	40	38
Marital status	married	married	married	single
Education	higher education	higher education	secondary school	secondary school
Employment	retired/self-employed	self-employed	part-time	unemployed
Health education	dentist	none	none	none
Household net income *	-	2400–3100 EUR	1800–2000 EUR	800–1000 EUR
Cigarette smoking	non-smoker	current smoker	non-smoker	current smoker
Other mental disorder	panic attacks	anxiety-depressive	anxiety, bulimia	none

Note. HL = health literacy, - = not given, * The household disposable net income per household is 1469 EUR per month in the Czech Republic [33].

**Table 2:** Overall scores (0–50) of the participants in the European Health Literacy Survey Questionnaire HLS-EU-Q47.

HL	Participant 1	Participant 2	Participant 3	Participant 4
	(Adequate)	(Adequate)	(Limited)	(Limited)
General health literacy	42	43	20	23
Healthcare	48	42	21	25
Disease prevention	37	46	22	16
Health promotion	40	43	18	25
Access health information	35	44	19	24
Understand health information	49	49	26	30
Appraise health information	43	39	14	11
Apply health information	41	42	23	23

Note. 0–25 = inadequate health literacy, >25–33 = problematic health literacy, >33–42 = sufficient health literacy, >42–50 = excellent health literacy.
